# Supplementary material for: Template switching in DNA replication can create and maintain RNA hairpins
Source: Proc Natl Acad Sci U S A. 2022 Jan 19;119(4):e2107005119. doi: 10.1073/pnas.2107005119 (PMC8794818; doi:10.1073/pnas.2107005119)
Supplement: Supplementary File [file pnas.2107005119.sapp.pdf]

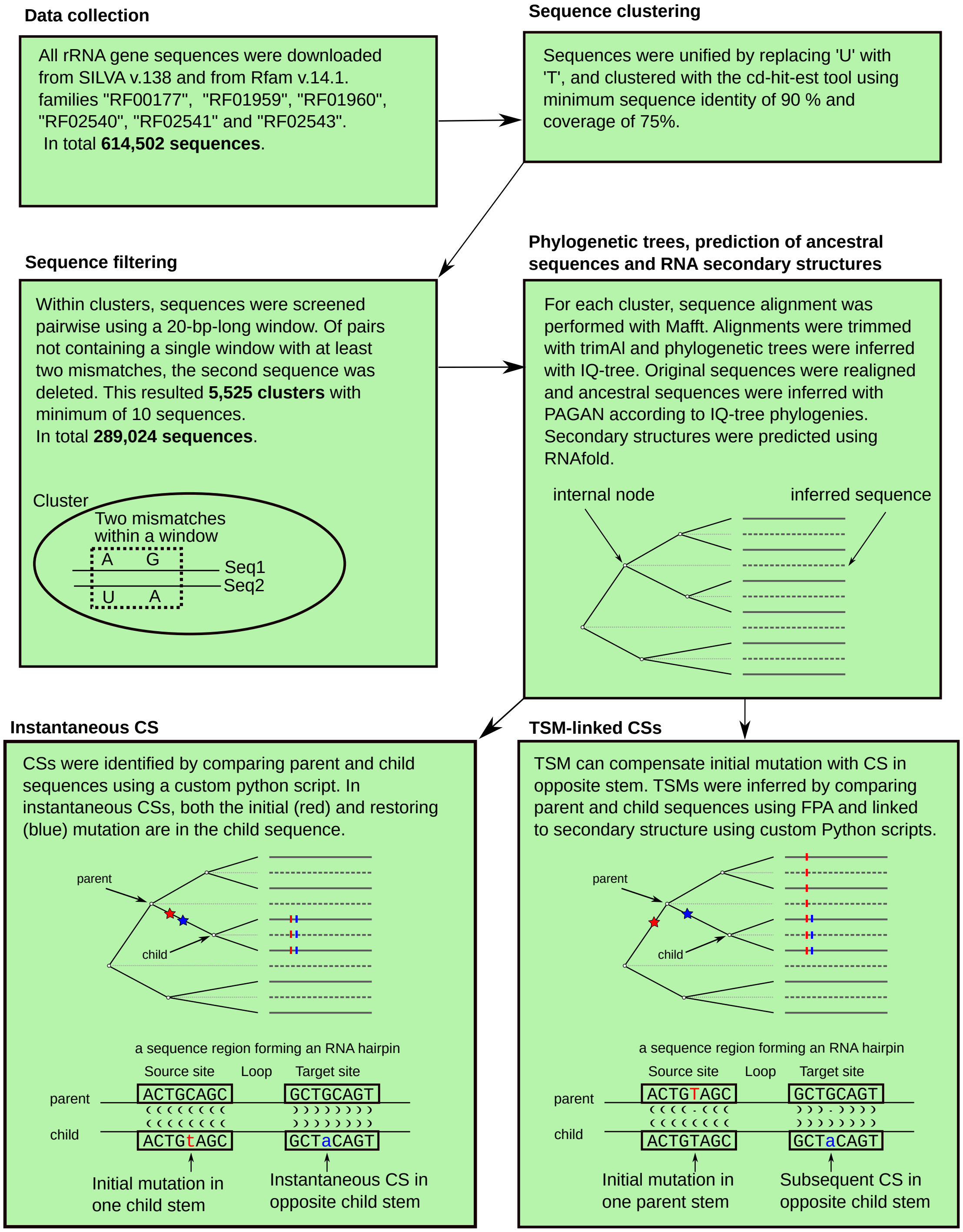

Fig. S1. The workflow for identifying instantaneous and two-step CMs from the data set.

PDBid (chain)	Identifier in this study	TSM target sequence	Predicted structure	Solved structure
6V3E (1)	12316_416	ACCAGG	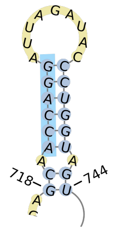 Genbank: GU356289.1	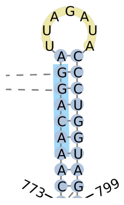
6V3E (1)	12316_427	*CCUGAG*	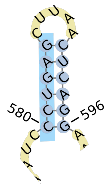 GenBank: JF232448.1	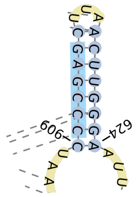
6OST (2)	12574_22	CUUUACUC	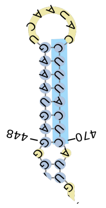 GenBank: EU467995.1	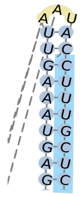
6OST (2)	12574_29	AAAGCAGC	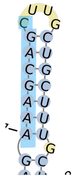 GenBank: KF842903.1	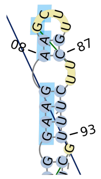

Fig. S2. Predicted and solved RNA structures for two-step CMs that share over 95% sequences identity with PDB RNA structures. Only one CM per PDB hairpin structure is shown. A TSM target sequence is highlighted in light blue.

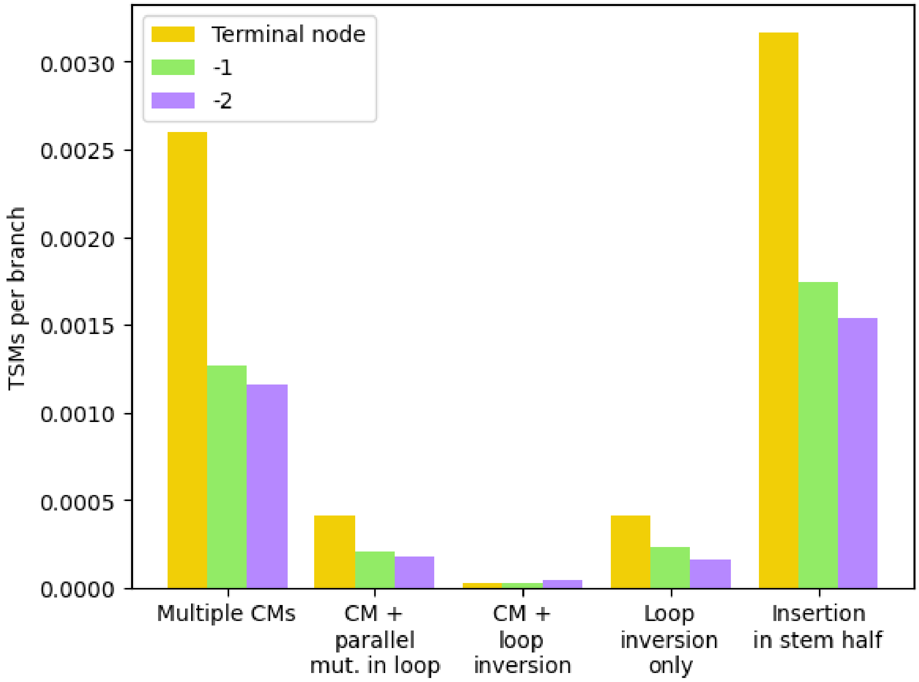

Fig. S3. Frequencies of CM-associated TSM patterns per branch. The frequency of events in terminal and sub-terminal (-1, -2) branches was calculated by dividing the number of inferred TSM events by the total number of branches on that tree level. Enrichment of TSM-like patterns causing structural changes in terminal branches suggests that many mutations are detrimental and eliminated by selection.

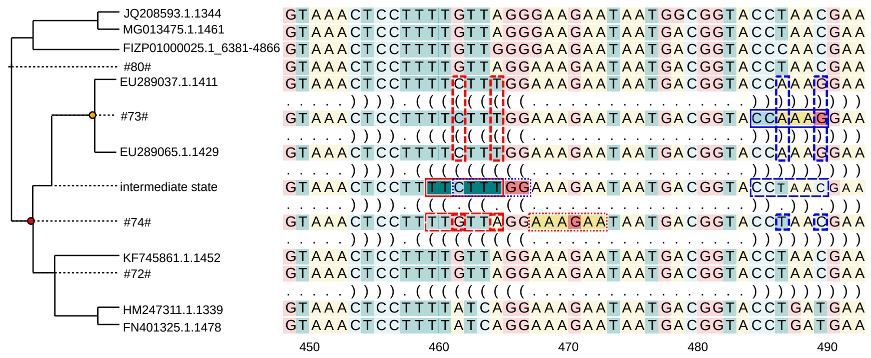

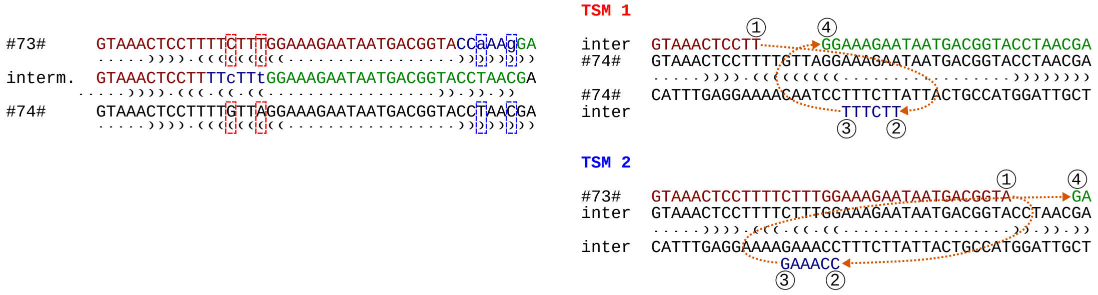

Fig. S4. Inferred history for a set of nematode rRNA sequences indicates a two-base instantaneous CM between nodes #74# and #73# where G-C and A-T are changing to C-G and T-A (red and blue dashed boxes). These can be explained with two TSM events (bottom right) and an intermediate state (top and bottom left). First, an asymmetric TSM ("TSM 1") breaks the base pairing by copying loop sequence into the stem (red boxes). Then, a symmetric TSM ("TSM 2") fixes the base pairing by copying overlapping sequence from one stem half to the other half (blue boxes). TSM source and target regions are framed with dotted and solid line, homologous regions with dashed line.

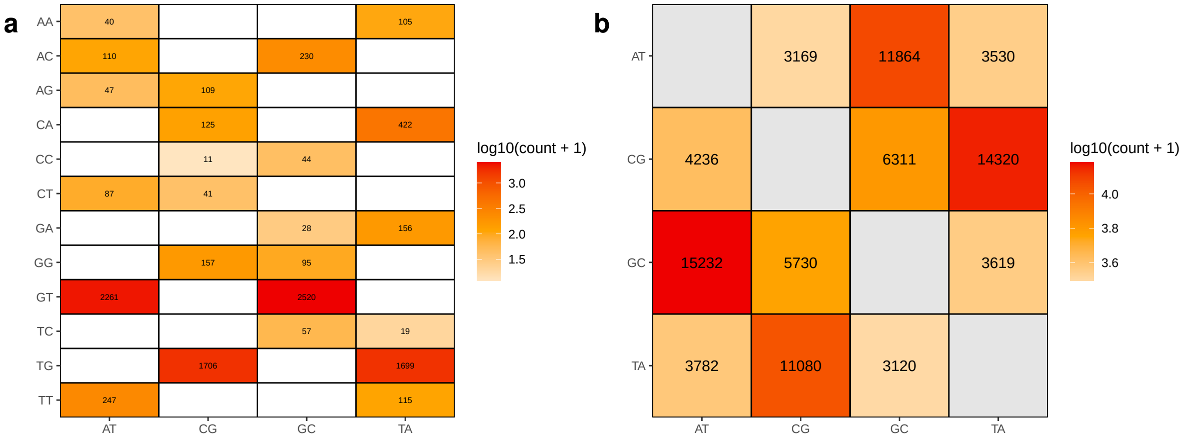

Fig. S5. Frequencies of base pairs involved in **a**) two-step and **b**) instantaneous compensating mutations. The ancestral state is shown on y axis.

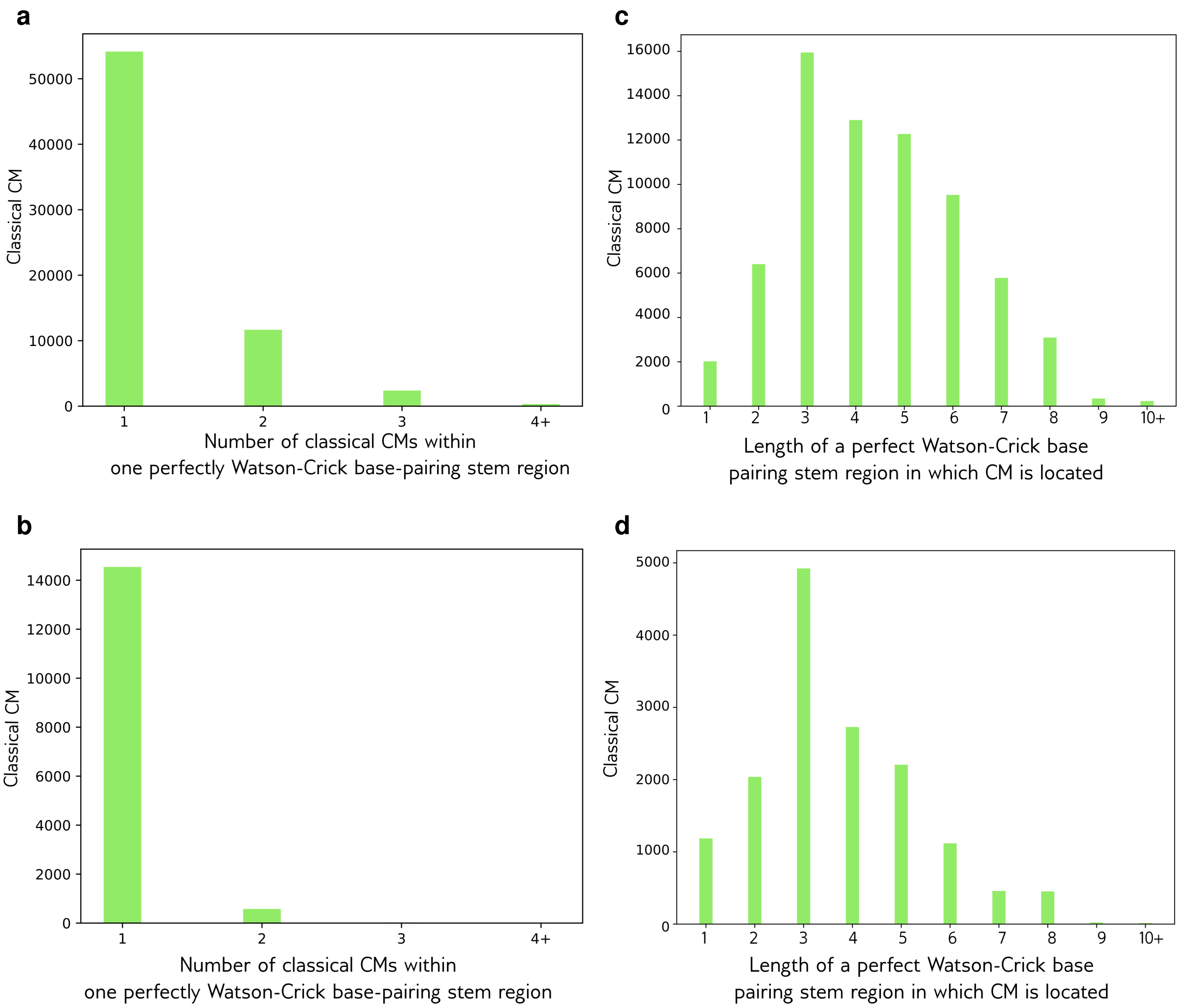

Fig. S6. Number of compensating mutations located within individual stem regions in **a**) terminal nodes and **b**) internal nodes. Lengths of perfect Watson-Crick base-pairing stem regions of instantaneous compensating mutation events in **c**) terminal nodes and **d**) internal nodes .

Table S1. Number of different types of TSM patterns consistent with two-step CM process in terminal and internal nodes of empirical and simulated data. The top and bottom halves show the results for TSM-like patterns with the ②-③ fragments of 6+ and 10+ bp, respectively. Significance values below 0.05 are emphasized in red. Correction factor (top) was used to account for the longer length of simulated data when computing the significance values.

Terminal (6+)	Empirical	Sim. 1	Sig. 1	Sim. 2	Sig. 2	Sim. 3	Sig. 3	Sim. 4	Sig. 4	Sim. 5	Sig. 5
Correction factor	1.000	1.104	NA	1.103	NA	1.101	NA	1.111	NA	1.100	NA
CM present	6,251	6,275	0.998	6,284	0.998	6,208	0.995	6,152	0.995	6,210	0.994
One CM only	5,890	6,031	1.000	6,025	1.000	5,957	0.999	5,915	0.999	5,971	0.999
Multiple CMs	310	193	0.000	184	0.000	181	0.000	180	0.000	177	0.000
CM + parall. mut. in loop	48	50	0.7521	73	0.997	67	0.987	52	0.8162	60	0.946
CM + loop inversion	3	1	0.178	2	0.367	3	0.547	5	0.801	2	0.366
Loop inversion only	48	10	0.000	14	0.000	16	0.000	12	0.000	5	0.000
Within either stem half	632	365	0.000	312	0.000	320	0.000	318	0.000	349	0.000
From loop to stem	141	65	0.000	82	0.001	85	0.002	75	0.000	89	0.005
Insertion in stem half	371	71	0.000	74	0.000	74	0.000	66	0.000	67	0.000

Internal (6+)	Empirical	Sim. 1	Sig. 1	Sim. 2	Sig. 2	Sim. 3	Sig. 3	Sim. 4	Sig. 4	Sim. 5	Sig. 5
Correction factor	1.000	1.105	NA	1.103	NA	1.102	NA	1.111	NA	1.100	NA
CM present	3,680	4,826	1.000	4,772	1.000	4,715	1.000	4,800	1.000	4,826	1.000
One CM only	3,516	4,687	1.000	4,649	1.000	4,589	1.000	4,670	1.000	4,692	1.000
Multiple CMs	140	94	0.015	97	0.023	89	0.005	90	0.007	88	0.004
CM + parall. mut. in loop	21	42	0.998	26	0.851	36	0.989	37	0.992	43	0.999
CM + loop inversion	3	3	0.549	0	0.0416	1	0.178	3	0.551	3	0.547
Loop inversion only	21	3	0.000	7	0.007	10	0.041	6	0.004	4	0.001
Within either stem half	376	187	0.000	244	0.000	223	0.000	207	0.000	209	0.000
From loop to stem	72	46	0.032	34	0.001	35	0.001	45	0.027	45	0.024
Insertion in stem half	196	51	0.000	46	0.000	46	0.000	52	0.000	60	0.000

Terminal (10+)	Empirical	Sim. 1	Sig. 1	Sim. 2	Sig. 2	Sim. 3	Sig. 3	Sim. 4	Sig. 4	Sim. 5	Sig. 5
Correction factor	1.000	1.105	NA	1.103	NA	1.101	NA	1.111	NA	1.100	NA
CM present	477	356	0.021	388	0.120	337	0.005	356	0.024	375	0.061
One CM only	385	330	0.279	363	0.664	325	0.136	326	0.0258	344	0.427
Multiple CMs	82	22	0.000	18	0.000	16	0.000	19	0.000	23	0.000
CM + parall. mut. in loop	7	4	0.223	6	0.460	4	0.225	8	0.677	7	0.571
CM + loop inversion	3	0	0.042	1	0.178	2	0.366	3	0.551	1	0.177
Loop inversion only	32	7	0.001	10	0.001	10	0.0025	8	0.000	3	0.000
Within either stem half	14	18	0.837	13	0.524	8	0.143	12	0.451	15	0.671
From loop to stem	10	11	0.665	17	0.937	10	0.582	10	0.589	15	0.882
Insertion in stem half	4	0	0.023	1	0.102	4	0.554	1	0.103	5	0.682

Internal (10+)	Empirical	Sim. 1	Sig. 1	Sim. 2	Sig. 2	Sim. 3	Sig. 3	Sim. 4	Sig. 4	Sim. 5	Sig. 5
Correction factor	1.000	1.105	NA	1.103	NA	1.102	NA	1.111	NA	1.100	NA
CM present	247	262	0.937	276	0.979	264	0.938	279	0.984	259	0.912
One CM only	220	252	0.987	268	0.998	250	0.981	266	0.997	246	0.973
Multiple CMs	22	5	0.002	6	0.004	9	0.023	7	0.008	11	0.057
CM + parall. mut. in loop	4	2	0.240	2	0.240	4	0.554	5	0.687	2	0.239
CM + loop inversion	1	3	0.858	0	0.159	1	0.527	1	0.530	0	0.159
Loop inversion only	13	2	0.003	5	0.043	5	0.043	5	0.044	1	0.001
Within either stem half	7	6	0.461	9	0.755	10	0.821	10	0.825	11	0.872
From loop to stem	7	5	0.342	6	0.460	6	0.459	6	0.467	7	0.571
Insertion in stem half	10	0	0.001	1	0.004	1	0.004	1	0.004	3	0.035

Abbreviations: Sim. = Simulated data set; Sig. = Significance (p-value)

Table S2. Number of cases in leaf nodes with one or more instantaneous CMs within a stem in empirical and simulated data, and with different lengths of Watson-Crick base pairing region.

Parallel CMs	Empirical data	Simulated data 1	Simulated data 2	Simulated data 3	Simulated data 4	Simulated data 5
1	54,155	3,206	3,317	3,186	3,255	3,295
2	11,666	22	26	27	32	19
3	2,380	0	1	1	0	1
4	298	0	0	0	0	0
5	30	0	0	0	0	0
6	4	0	0	0	0	0

Length of W-C pairing	Empirical data	Simulated data 1	Simulated data 2	Simulated data 3	Simulated data 4	Simulated data 5
1	2,032	154	182	197	164	180
2	6,406	482	533	482	518	549
3	15,949	840	883	894	850	853
4	12,902	781	772	734	785	789
5	12,274	488	512	456	515	490
6	9,527	286	269	290	281	281
7	5,783	133	120	106	116	114
8	3,095	48	52	43	38	43
9	344	9	12	10	15	12
10+	230	7	9	0	5	4

Table S3. One hundred most frequent RNA hairpin loops in terminal node sequences. For each sequence, the counts of forward and reverse complement sequences in the full data and their ratio are shown.

Top 1-50			
	Counts		
Sequence	Forward	Rev. comp.	Ratio
GAAA	304530	3089	98.59
GCAA	280342	516	543.3
TACG	175986	764	230.35
AGACA	116960	6693	17.47
ATTAGATAC	114835	0	NA
GAGA	113134	322	351.35
TTCG	95385	2600	36.69
GAAG	93507	1934	48.35
AGCC	90198	110	819.98
TGCAACTC	88008	0	NA
CTCAA	83168	0	NA
GTGAAATG	77604	0	NA
TTAAGT	69794	36	1938.72
GCGA	68284	1447	47.19
GTAACGG	59105	0	NA
ACAA	56090	3233	17.35
GTCAA	48956	638	76.73
GCAT	48804	899	54.29
GACAA	44615	71	628.38
GATAA	38193	513	74.45
CTTAA	38154	2	19077
TAAC	35674	2690	13.26
GTGA	35270	3361	10.49
GGAA	34826	9283	3.75
GTAATGG	33875	0	NA
TAAA	33616	7252	4.64
GTAA	33337	5326	6.26
TGAGAGGAT	30680	0	NA
TCGT	30642	369	83.04
GCT	30435	857	35.51
CTTG	27722	3383	8.19
GTGAAA	27569	42	656.4
AAGCAACG	26930	0	NA
TCAAAT	25347	149	170.11
GACGTCAA	24107	0	NA
CAA	23378	3287	7.11
GTAAAGG	22729	5	4545.8
GGGAA	21387	48	445.56
AAA	18716	12072	1.55
TAGTAATC	17504	11	1591.27
TTC	17267	6902	2.5
TGAAAT	17021	39	436.44
TGAAATT	14856	2	7428
TACA	14572	1051	13.86
TGCAACCC	13916	0	NA
ATCAGAATG	13850	0	NA
GGTGGA	13408	1	13408
TAT	13085	5603	2.34
GTAATA	12911	20	645.55
ATGCAACG	12559	0	NA

Top 51-100			
	Counts		
Sequence	Forward	Rev. comp.	Ratio
TTT	12072	18716	0.65
ATTTA	11914	685	17.39
GAGG	11873	99	119.93
TGAGAAAC	11735	0	NA
AATT	11430	11430	1
GTGGAATG	11230	0	NA
AAT	11204	4031	2.78
ATCAGCATG	11034	0	NA
TTAT	10882	7033	1.55
CTCG	10829	1327	8.16
TTTT	10817	6390	1.69
TGAGAGGAC	10534	2	5267
ATTAA	10468	3514	2.98
AGATA	10261	39	263.1
AAAC	10228	2170	4.71
CATCA	10174	0	NA
AAAG	9961	657	15.16
TGAA	9737	1010	9.64
GTTAA	9516	795	11.97
TCCG	9507	353	26.93
AATTA	9502	2036	4.67
GCCAA	9341	95	98.33
AACGA	9288	191	48.63
TTCC	9283	34826	0.27
TTAA	9263	9263	1
TCT	9256	2826	3.28
AACG	9204	114	80.74
GCTCAA	9033	1	9033
TCG	9005	2338	3.85
GAGTA	8988	73	123.12
AAC	8662	3112	2.78
AAATG	8386	40	209.65
GTAATG	7923	11	720.27
AATG	7673	120	63.94
CGTG	7615	4631	1.64
GAAGATAATGACGGTA	7596	0	NA
TTTA	7252	33616	0.22
AGGAA	7107	33	215.36
GAAGGCG	7084	0	NA
ATAA	7033	10882	0.65
ATTCA	7020	483	14.53
AATTCG	6954	9	772.67
ATAATGA	6911	345	20.03
GAA	6902	17267	0.4
ACA	6901	2047	3.37
TTAGATGTTCT	6729	0	NA
TAA	6723	5338	1.26
TGTCT	6693	116960	0.06
TTTAGT	6637	72	92.18
TTAATAC	6604	50	132.08

Table S4. Parameters for FPA and filtering of TSM results.

FPA parameter	Value
--min_length	6
--scan_window_limit	1
--force_overlap	True
--clean_rna	True
--iupac	True

Filtering criteria	Value
identity-upstream	> 0.9
identity-②-③-area	> 0.9
identity-downstream	> 0.9
full-identity-with-tsm	> full-identity-original
# nonidentities† in tsm-②-③-area	0
# nonidentities† in original-②-③-area	> 0

†includes insertions, deletions and mismatches
